# Upper Limb Rehabilitation Robot Powered by PAMs Cooperates with FES Arrays to Realize Reach-to-Grasp Trainings

**DOI:** 10.1155/2017/1282934

**Published:** 2017-06-15

**Authors:** Xikai Tu, Hualin Han, Jian Huang, Jian Li, Chen Su, Xiaobo Jiang, Jiping He

**Affiliations:** ^1^School of Industrial Design, Hubei University of Technology, Wuhan 430068, China; ^2^School of Automation, Huazhong University of Science and Technology, Wuhan 430074, China; ^3^School of Mechanical Engineering, Hubei University of Technology, Wuhan 430068, China; ^4^Advanced Innovation Center for Intelligent Robots and Systems, Beijing Institute of Technology, Beijing 100081, China; ^5^Arizona State University, Tempe, AZ 85287, USA

## Abstract

The reach-to-grasp activities play an important role in our daily lives. The developed RUPERT for stroke patients with high stiffness in arm flexor muscles is a low-cost lightweight portable exoskeleton rehabilitation robot whose joints are unidirectionally actuated by pneumatic artificial muscles (PAMs). In order to expand the useful range of RUPERT especially for patients with flaccid paralysis, functional electrical stimulation (FES) is taken to activate paralyzed arm muscles. As both the exoskeleton robot driven by PAMs and the neuromuscular skeletal system under FES possess the highly nonlinear and time-varying characteristics, iterative learning control (ILC) is studied and is taken to control this newly designed hybrid rehabilitation system for reaching trainings. Hand function rehabilitation refers to grasping. Because of tiny finger muscles, grasping and releasing are realized by FES array electrodes and matrix scan method. By using the surface electromyography (EMG) technique, the subject's active intent is identified. The upper limb rehabilitation robot powered by PAMs cooperates with FES arrays to realize active reach-to-grasp trainings, which was verified through experiments.

## 1. Introduction

Nowadays, the population of patients with limb motor dysfunction is increasing, which is caused by nerve injuries associated with stroke, traumatic brain injury, or multiple sclerosis. Particularly, the prevalence rate of stroke in China is increasing rapidly. Stroke survivors with various degrees of motor dysfunction not only endure inconvenience of the daily lives but also feel great psychological pressure, in addition to economic burden on the family and society. Many types of rehabilitation robots have been developed to assist rehabilitation in individuals with stroke [1–9]. In order to help stroke patients to receive intensive rehabilitation trainings as much as possible, cost-efficient portable rehabilitation equipment used in the community or home should be developed for patients after discharge, which would be a major improvement of limb rehabilitation.

Many stroke rehabilitation experiments show a positive role in using FES for recovery of motor function. FES is a method for activating sensory-motor systems by delivering electrical charge in the form of bursts of electrical pulses. By surface electrodes, FES stimulates motor or sensory nerves of muscles and facilitates motor rehabilitation and function reconstruction. Wu et al. [10] adopted a hybrid method of combining bilateral arm training with FES in patients poststroke to improve hand function, and a linear guide platform with FES feedback control is developed to execute the training of bilateral reaching movements. A robotic workstation for stroke rehabilitation of upper extremity using FES is developed by Freeman et al. [11]. They use voluntary control with the addition of electrical stimulation applied to muscles in the impaired shoulder and arm. FES can also realize the inhibition of abnormal reflexes and induce active movements [12]. Freeman et al. [13–15] in the University of Southampton have developed a portable upper limb exoskeleton system for reaching rehabilitation trainings. The rehabilitation system is composed of a FES stimulator and the passive Armeo Spring [16]. It uses the spring force to compensate for the gravity of the patient's upper limb and uses FES to activate paralyzed muscles to produce driving power. Rehabilitation training is a kind of continuous training with a certain intensity, but it will not be continuous due to muscle fatigue caused by electrical stimulation. The power of this system is all generated from FES stimulating the muscle, so training time and intensity of training will depend on whether the muscle state is fatigue or not.

RUPERT, a portable upper limb exoskeleton rehabilitation robot, is developed by Arizona State University. The system with five degrees of freedom (DOFs) is activated by low-cost PAMs and controlled by adaptive sensory feedback control algorithms for smooth- and safe-guarded movements during the task-oriented training. The unique features of the proposed robotic system are that it (1) is anchored on each user's trunk and aligned at the shoulder of the trained arm; (2) generates unidirectional assistive pulling force in each joint to encourage active participation of the user during each movement; (3) provides gravity compensation only if the user is too weak; and (4) evaluates the effectiveness of therapy by performance analysis, which includes kinematic criteria and users' effort. The new design makes the proposed robotic device portable for the user and can be used in various positions (sitting or standing) and different locations. By using less actuators, the weight and cost of the robot have been significantly reduced. The therapeutic benefits of the robot are not limited since antigravity tasks can be carried out by the motion control system which adapts to specific gravity compensation. In comparison with our previous studies [6, 17-18], this research mainly focuses on the safety and feasibility of our latest robotic arm, which has one more DOF of humeral internal/external rotation for enlarging the reaching space. More stroke patients were enrolled, and further biometric analyses were performed including clinical laboratory therapy sessions and in-home therapy sessions for the purpose of enabling frequent training at home. Despite all this, RUPERT with one way actuator of the joint is not suitable for stroke patients with weak muscles in the flaccid paralysis period. In order to expand the range of RUPERT rehabilitation application including reaching exercises for ordinary patients with flaccid paralysis, FES is used to activate paralyzed muscles. FES induced muscle force, and a pneumatic muscle pull force is a new kind of combination actuation, which can produce muscle torque and compensate the drawbacks of RUPERT. They cooperate together and realize the robotic joint two-way movement. Our proposed hybrid system in this research can allow patients to receive more lasting endurance rehabilitation trainings than the system developed by C.T. Freeman et al. As both the exoskeleton robot driven by PAMs and neuromuscular skeletal system under FES possess the highly nonlinear and time-varying characteristics, which add control difficulty to the hybrid dynamic system, ILC is studied and taken to control this newly designed hybrid rehabilitation system to realize repetitive task trainings. The transfer of ILC to rehabilitation is based on the patient making repeated attempts to complete a task, such as reaching out over a table top to an object.

The ability of grasping and releasing the object plays an important role in our daily lives. Most patients with stroke suffer from the hand dysfunction, the symptoms of which are that finger flexor muscle tone is high and patients cannot open their own hands actively. The hand rehabilitation includes two kinds of intelligent strategies: robot-assisted and FES. Heo et al. summarized the existing multi-DOF hand rehabilitation exoskeletons [19]. The number of the hand's joints is up to 22 DOF, which will make the mechanical and electrical designs of the hand exoskeleton very complicated. As stroke patients' hands show the phenomenon of abnormal nerve reflex, the rehabilitation exoskeleton is hard for patients to wear, even patients experiencing the secondary damage in the process of putting it on. FES can activate paralyzed muscles to produce joint movement through stimulus pulses conducted by use of surface-adhesive electrodes, but the precise finger joint movement by FES is not realized and therefore it is difficult to produce FES-induced functional grasping and releasing. The reasons are that the shapes of ordinary self-adhesive electrodes are bigger and their stimulus selectivity is not enough and yet it can activate many finger muscles at the same time. Westerveld et al. [20] invented an artificial way to paste small pad electrodes above the motor points of finger flexors and extensors and implemented the hand grasping and releasing with the help of FES and model predict control. The shortcomings of this method are that it takes much time of the therapist to place small electrodes accurately on the corresponding motor points. Malešević et al. [21] developed a 4 × 4 electrode array, which can achieve the intelligent trainings of hand grasping and releasing by virtue of FES, but it cannot implement reach-to-grasp trainings. In order to realize reach to grasp trainings, Westerveld et al. added a 3-DOF end-effector rehabilitation robot to the FES system with pad electrode [22]. They used robot and FES to achieve reaching and grasping, respectively [23], but this end-effector rehabilitation system guided the patient's upper limb movement only through his/her hand but the shoulder, elbow, and wrist joints could not be rehabilitated individually. RUPERT upper limb rehabilitation exoskeleton integrated with FES can overcome the above shortcomings by the search algorithm. The surface electrode array is composed of many small electrodes arranged in matrix 4 × 6 form, which can realize each finger selective stimulation. This setup can solve the time-consuming problem of self-adhesive electrode placement while RUPERT can realize the multijoint coordination trainings.

Active training involves motion intention recognition, and now these sensors most widely used include two categories: electromechanical and bioelectrical. Electromechanical sensors mainly include position and force/torque ones, and this kind of sensors has electromagnetic mechanical time delay, especially expensive high-performance multiaxis force/torque sensors. Bioelectric sensors collect biological signals such as ECG, EMG, and EEG, and the time delay of this kind is shorter than the previous kind. EMG occurs 20–30 milliseconds ahead of muscles producing joint movement. Many rehabilitation devices use surface EMG to extract biological information as a way of identifying human motion intention. It is also used in the intention-based FES to actively activate the muscles to produce movement, but surface EMG is buried in stimulus artifact and induced muscle response (M wave). Comb filter and blanking window methods are used to extract intention information for the active intent.

In this paper, it introduces that upper limb rehabilitation robot powered by PAMs cooperates with FES arrays to realize active reach-to-grasp trainings for stroke patients. In Section 2, the dynamic models of a pneumatic muscle and FES-induced muscle are built for reaching trainings. In Section 3, the subject's active intent is identified using EMG and grasping and releasing are realized by FES array electrodes.  Section 4 introduces the ILC control strategy and its practical application to reach-to-grasp trainings by virtue of robot and FES.  Section 5 reports the experimental results of PAMs in cooperation with FES arrays to realize active reach-to-grasp trainings. Conclusion and future work are shown in Section 6.

## 2. Dynamic Models of PAMs and FES Muscle for Reaching Trainings

### 2.1. The Modeling and Identification of RUPERT

**Figure 1:**
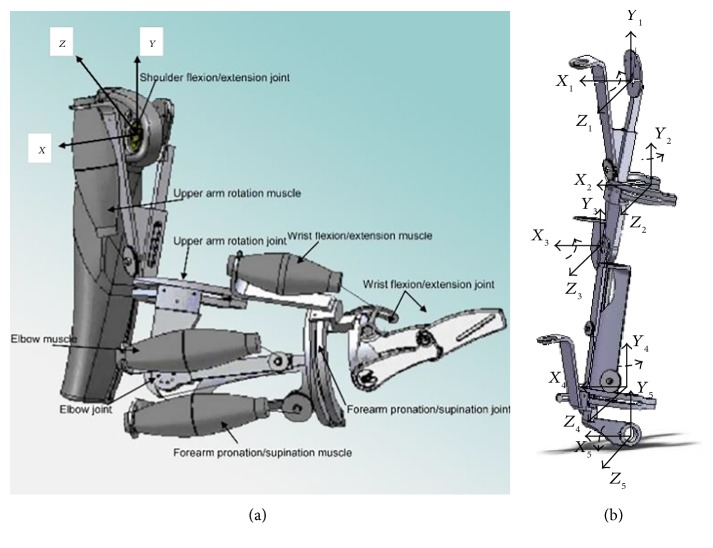
(a) The assembly drawing of the 5-DOF upper limb exoskeleton of the RUPERT and (b) the coordinate system diagram of RUPERT [6].

**Figure 2:**
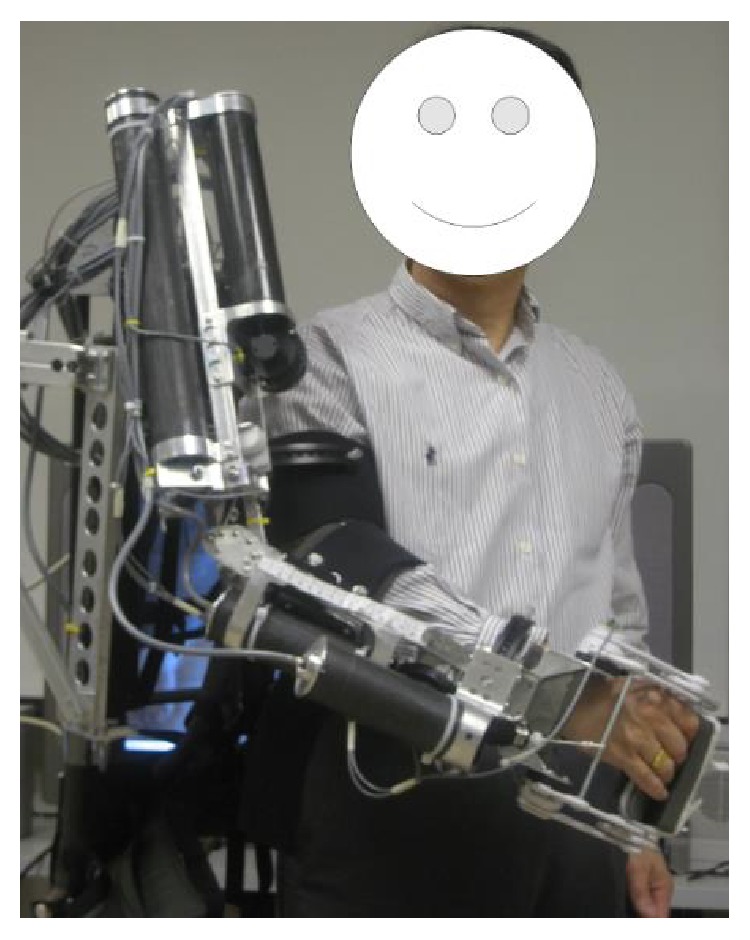
The diagram of the subject wearing the 5-DOF RUPERT [6].

The RUPERT upper limb rehabilitation robot has 5 DOFs: shoulder flexion/extension, humeral internal/external rotation, elbow flexion/extension, forearm pronation/supination, and wrist flexion/extension shown in the Figure 1(a).  Figure 1(b) shows the mechanical design of the RUPERT robot. For each DOF, a pneumatic muscle is used as a unidirectional actuator to generate a joint pulling force. This accords with the stroke patients' symptoms and that means the muscle is in a condition of high muscular tension while flexor muscles will produce involuntary contraction. As FES can stimulate paralyzed muscles to move against a PM-driving direction, RUPERT can achieve two-way joint movement with the help of FES, which enables more patients to use RUPERT to do rehabilitation trainings in different recovering phases. The depiction of a subject wearing the RUPERT robot is shown in Figure 2.

(1) $$\begin{array}{c} \bar{M}\left(\theta \right)\ddot{\theta }+\bar{B}\left(\dot{\theta }\right)\dot{\theta }+\bar{K}\left(\theta \right)\theta +\bar{G}\left(\theta \right)+\Delta \bar{\tau }={\bar{\tau }}_{p}-{\bar{\tau }}_{FES}. \end{array}$$

The hybrid dynamic system of RUPERT exoskeleton and FES neuromuscular model is shown in (1) and in which *θ*,$\dot{\theta }$,$\ddot{\theta }\in R^{5}$ are joint angle, angular velocity, and angular acceleration of 5-DOF RUPERT, respectively. $\bar{M}\left(\theta \right)\in R^{5\times 5}$ is symmetric positive definite inertia matrix, and $\bar{B}\left(\overset{˙}{\theta }\right)$,$\bar{K}\left(\theta \right)\in R^{5\times 5}$ are damping matrix and stiffness matrix, respectively. $\bar{G}\left(\theta \right)\in R^{5}$ is the gravity moment. Based on the previous work [17, 18, 24, 25], it is known that $\bar{M}\left(\theta \right)$, $\bar{B}\left(\dot{\theta }\right)$, and $\bar{K}\left(\theta \right)$ are diagonal matrix and $\bar{B}\left(\dot{\theta }\right)$ and $\bar{K}\left(\theta \right)$ are set to a constant value shown in (2). The values of $\bar{M}\left(\theta \right)$ and $\bar{G}\left(\theta \right)$ vary according to the different subjects by use of the specific calculation method according to the literature [17]. $\Delta \bar{\tau }$ is torque generated by patients' muscle forces and other disturbances. ${\bar{\tau }}_{p}$ is the torque generated by pneumatic muscle, and ${\bar{\tau }}_{FES}$is torque generated by neuromuscular electrical stimulation. The dynamic models of pneumatic muscle play an important role in this hybrid combination. So this chapter introduces the modeling and identification of pneumatic muscle.

(2) $$\begin{array}{c} \begin{array}{l} \bar{B}\left(\dot{\theta }\right)=\mathrm{diag}\left(\left[0.01,0.015,0.03,0.02,0.02\right]\right)\\ \bar{K}\left(\theta \right)=\mathrm{diag}\left(\left[0.005,0.02,0.01,0.005,0.01\right]\right). \end{array} \end{array}$$

By virtue of the physical model, the controlling of the pneumatic system would become more complex, as some parameters are not easy to be detected. After linear simplification, damping-related items are omitted, which are not conducive to the relatively rapid real-time control of the lower limb gait. The phenomenon model is taken from the external observation, which is usually represented by a mass-stiffness-damping dynamic system. This kind of model is also called the three-element model of pneumatic muscle, and its equations can be described as shown in the following:

(3) $$\begin{array}{c} M\left(\ddot{x}+g\right)+B\left(P\right)\dot{x}+K\left(P\right)x=F\left(P\right)\\ B\left(P\right)=B_{0}+B_{1}P\\ K\left(P\right)=K_{0}+K_{1}P\;\\ F\left(P\right)=F_{0}+F_{1}P\\ B\left(P\right)=B_{0i}+B_{1i}P\left(inflation\right)\\ B\left(P\right)=B_{0d}+B_{1d}P\;\left(deflation\right). \end{array}$$

**Figure 3:**
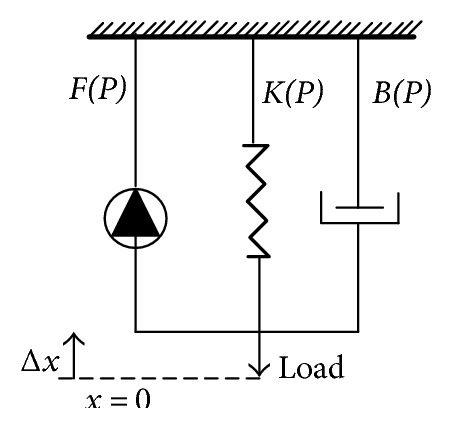
Three-element model of pneumatic muscle.

**Figure 4:**
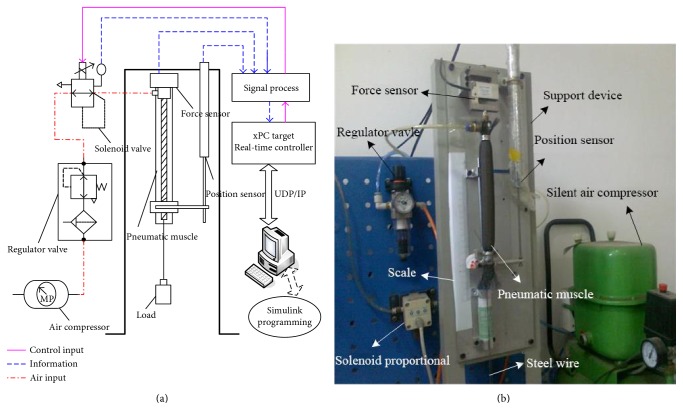
The platform for modeling the characteristics of pneumatic muscle, (a) the pneumatic circuit and control platform, and (b) the experimental device for modeling pneumatic muscle.

Three elements include inertia *M*, damping *B*, and stiffness *K* shown in Figure 3. The only input control variable is pneumatic pressure *P*, and *F* (*P*) is active contraction force. Damping B has different values according to the process of inflation and deflation, respectively. $B\left(P\right)\dot{x}$ is the viscous force impeding the pneumatic muscle movement itself, and *K*(*P*)*x* is the spring force impeding the pneumatic muscle shortening. $M\left(\ddot{x}+g\right)$ is the driving force for the load, of which *M* is the load mass, *g* is the gravity acceleration, and *x* is the axial contraction length of the pneumatic muscle. *x* = 0 is marked as the initial position of the pneumatic muscle in a completely bleeding state. The contraction coefficient and stiffness coefficient are obtained by using different pressures and least square method (LSM) through the static force balance experiments. Damping system is obtained through the static disturbance experiment, and for their specific identification process, please refer to the literature [26]. Pneumatic muscle experimental platform is shown in Figure 4 while the identification results are shown in Table 1.

### 2.2. Modeling and Identification of FES Muscle

Neuromuscular electrical stimulation models are widely used in various fields of researches, which can explore the characteristics of isometric and nonisometric contraction of muscles. In the case of nonisometric contraction, the force produced by the muscle is not only related to the length of contraction, but also the rate of muscle contraction. If the muscle lies in the condition of equal length changes such as elongation or shortening in the case of isometric contraction, the muscle produces the maximum contraction force and then the maximum muscle contraction force will be reduced. Hill model is the most commonly used model for muscle modeling, by use of mass-spring-damping to describe the dynamic behavior of muscle. Durfee model [27] is expanded on the basis of the Hill muscle model.

**Figure 5:**
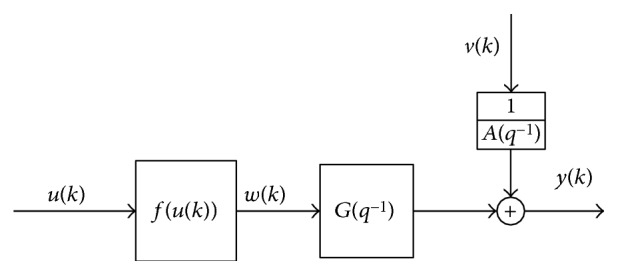
Discrete-time Hammerstein model.

*u*(*k*) is the input variable of electrical stimulation signal, and *k* is the *k*th sampling. *f*(*u*(*k*)) is the “static nonlinear” function of the discrete-time Hammerstein model shown in Figure 5(), and linear dynamic function is *G*(*q*^−1^). *q*^−1^ is delay factor, and *m* and *n* are the poles and zeroes of the transfer function *G*(*q*^−1^), respectively. *d* is the sample number of time delay. *v*(*k*) is disturbance, and *y*(*k*) is the output of the neuromuscular electrical stimulation of muscle force or torque. Nonlinear function *f*(*u*(*k*)) is the cubic spline function; *u*_1_,*u*_2_,*u*_3_,…,*u*_*l*_ are cubic spline interpolation points shown in the following [28]:

(4) $$\begin{array}{c} G\left(q^{-1}\right)=\frac{B\left(q^{-1}\right)}{A\left(q^{-1}\right)}=\frac{q^{-d}\left(b_{0}+b_{1}q^{-1}+\cdots +b_{n}q^{-n}\right)}{1+a_{1}q^{-1}+\cdots +a_{m}q^{-m}} \end{array}$$

(5) $$\begin{array}{c} \theta_{G}=\left[\begin{array}{l} \theta_{a}\\ \theta_{b} \end{array}\right]={\left[a_{1}\;\cdots \;a_{1}\;b_{0}\;b_{1}\;\cdots \;b_{n}\right]}^{T} \end{array}$$

(6) $$\begin{array}{c} \begin{array}{c} f\left(u\left(k\right)\right)=\overset{l-2}{\underset{i=1}{\sum }}c_{i}\left|u\left(k\right)-u_{i+1}\left(k\right)\right|{}^{3}+c_{l-1}+c_{l}u\left(k\right)+c_{l+1}u^{2}\left(k\right)+c_{l+2}u^{3}\left(k\right),u_{\min}=u_{1}<u_{2}<u_{3}<\cdots <u_{l}=u_{\max} \end{array} \end{array}$$

(7) $$\begin{array}{c} \theta_{f}={\left[c_{1}\;c_{2}\;\cdots \;c_{l\;+\;2}\right]}^{T} \end{array}$$

(8) $$\begin{array}{c} \theta =\left[\begin{array}{l} \theta_{G}\\ \theta_{f} \end{array}\right] \end{array}$$

(9) $$\begin{array}{c} {\left\|v\right\|}_{2}^{2}=\overset{N}{\underset{k=1}{\sum }}v^{2}\left(k\right). \end{array}$$

Therefore, (9) can be regarded as the problem of least square shown in (10) to (11). Hammerstein structure nonlinear function and linear system parameter identification are taken by the use of an iterative algorithm using the ARX model.

(10) $$\begin{array}{c} \underset{\theta_{f}}{\arg\;\min}{\left\|Y_{f}\left(y,{\hat{\theta }}_{a}\right)-\Phi_{f}\left(u,{\hat{\theta }}_{b}\right)\theta_{f}\right\|}_{2} \end{array}$$

(11) $$\begin{array}{c} {\hat{\theta }}_{G}=\Phi_{G}{\left(u,y,{\hat{\theta }}_{f}\right)}^{T}\Phi_{G}{\left(u,y,{\hat{\theta }}_{f}\right)}^{-1}\Phi_{G}{\left(u,y,{\hat{\theta }}_{f}\right)}^{T}Y^{\prime }. \end{array}$$

The surface electrical stimulator (Hasomed, Rehastim2) is a constant current source with eight stimulus channels. Real-time control can be realized based on the ScienceMode2 communication protocol by the use of RS232 serial port with Simulink xPC Target.

The process of generating test data is called TR (triangular ramp). The value of pulse width is linear from 0 to 350*μs* and then back to 0 value. Its range has been distributed while the stimulus frequency is 20 Hz, and the stimulus amplitude is 20 mA. Nonlinear (6) of this proposed electrical stimulation model is rewritten as (12). Neuromuscular electrical stimulation dynamic contraction process can be expressed with the two order system [28], so the parameter values of linear expression (4) are *d* = 1, *n* = 1, *m* = 2, respectively. The electrical stimulation model parameters of the biceps are shown in Table 2, and the functional equation of identification is shown in (13) to (14). Consider

(12) $$\begin{array}{c} f\left(u\right)=\beta_{1}\left|u-150\right|{}^{3}+\beta_{2}+\beta_{3}u+\beta_{4}u^{2}+\beta_{5}u^{3} \end{array}$$

(13) $$\begin{array}{c} f\left(u\right)=-0.0294+0.0023u-6.98\times 10^{-6}u^{2}+1.56\times 10^{-8}u^{3}+2.41\times 10^{-8}\left|u-150\right|{}^{3} \end{array}$$

(14) $$\begin{array}{c} G\left(q^{-1}\right)=\frac{q^{-1}\left(1-0.364q^{-1}\right)}{1-1.21q^{-1}+0.117q^{-2}}. \end{array}$$

## 3. EMG Triggered Active Grasping and Releasing Trainings

### 3.1. Surface Array Electrodes Used in the Grasping and Releasing

**Figure 6:**
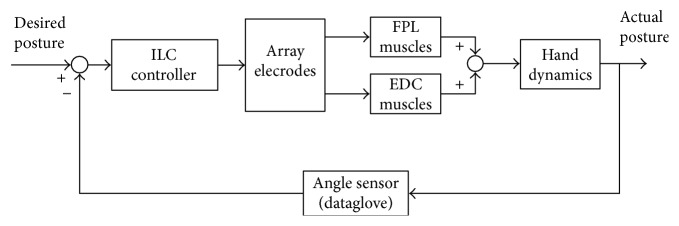
Control diagram of FES array electrodes to realize hand grasping and releasing.

**Figure 7:**
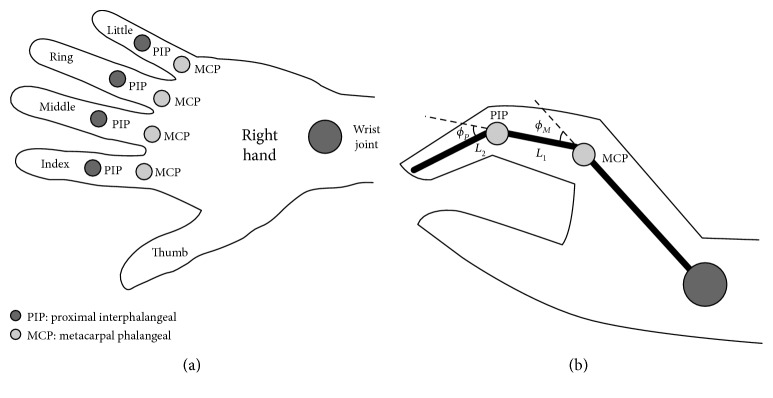
Schematic diagram of finger joint and angle.

**Figure 8:**
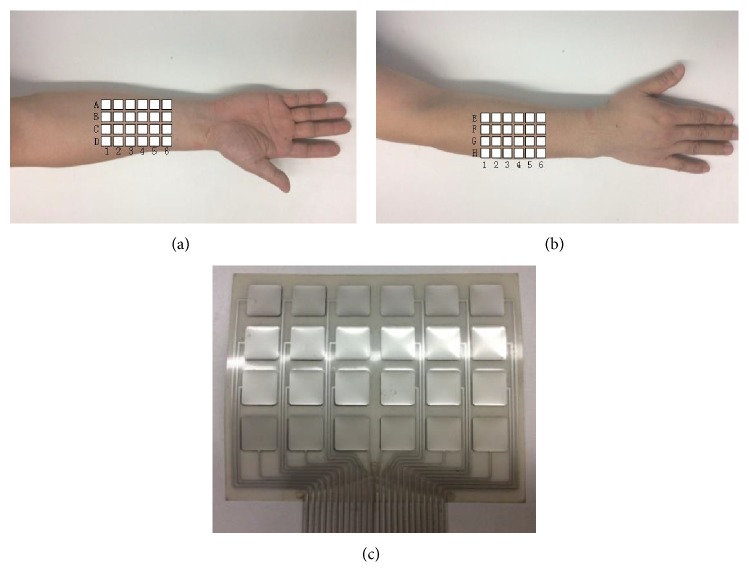
(a) Array electrode for FPL, (b) array electrode for EDC, and (c) array electrode prototype.

Surface finger muscles related with grasping and releasing include flexor pollicis longus (FPL), extensor digitorum communis (EDC), and thumb thenar muscle. Grasping is generally divided into two categories: power grasping and precision grip. To test the effectiveness of grasping and releasing by using the exoskeleton and FES, “power grasping” under electrical stimulation is relatively simple and taken as the research paradigm. To be simplified, flexor pollicis longus (FPL) and extensor digitorum communis (EDC) are selected as the stimulus objects shown in Figure 6. 42% of hand movement only needs four fingers, including the index finger, middle finger, ring finger, and little finger. Metacarpal phalangeal (MCP) is the metacarpophalangeal joint, and proximal interphalangeal (PIP) is the proximal interphalangeal joint. *ϕ*_*kM*_ is the angle of the palm and finger joint while *ϕ*_*kP*_ is the proximal interphalangeal joint angle, and *k* = 1, 2, 3, 4 represent the index finger, middle finger, ring finger, and little finger, respectively, as shown in Figure 7. 4 × 6 array electrodes in Figure 8were used while stimulation system adopted German Rehastim2, which used the Omron G3MB solid-state relay with switch frequency 5 kHz. MCP and PIP joint angles were measured by using Cyberglove.

(15) $$\begin{array}{c} RMS=\sqrt{\frac{1}{8}\overset{4}{\underset{k=1}{\sum }}\left[{\left(\phi_{dM}\left(t\right)-\phi_{kM}\left(t\right)\right)}^{2}+{\left(\phi_{dP}\left(t\right)-\phi_{kP}\left(t\right)\right)}^{2}\right]}. \end{array}$$

**Figure 9:**
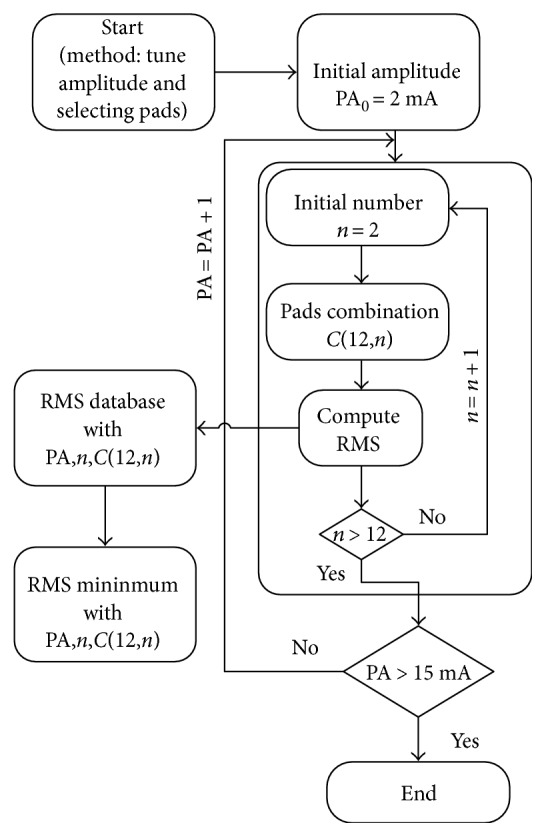
Block diagram of array electrode matrix scanning method.

The desired hand gesture was realized by matrix scanning method applied to array electrodes. The joint angle errors were kept within the plus or minus 3 degrees. Electrical stimulation frequency was 20 Hz, and stimulating pulse width 350 was constant. Stimulating pulse width 0–15 mA was regarded as the stimulus variable, and *u* was the electrical stimulation amplitude. Root mean square error RMS in (15) was taken as a performance optimization goal. When RMS was the smallest, the corresponding combination of electrode array targets was selected by trials and errors. The range of stimulus amplitude was from 2 mA to 15 mA while electrode array target number was from *n* = 2 to *n* = 12, as shown in Figure 9.

### 3.2. Real-Time Intention Extraction of Surface EMG under FES

**Figure 10:**
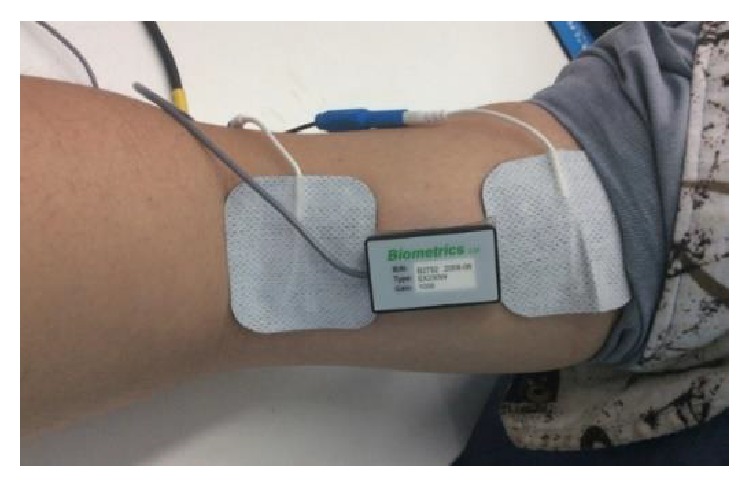
The picture of intention EMG extraction under electrical stimulation.

Intention-based EMG can be taken as the trigger signal of robot and FES, but it is contaminated by FES stimulus artifact in this research. A strategy was developed that real-time intention surface EMG was extracted from FES stimulus signals. Surface EMG sensor was using the model SX230 of Biometrics Corporation. Simulink xPC target system was used, as shown in Figure 10.

(16) $$\begin{array}{c} y\left(k\right)=\frac{x\left(k\right)-x\left(k-N_{s}\right)}{\sqrt{2}}. \end{array}$$

Electrical stimulation artifact was detected using Simulink Comb filter [29], shown in (16) to (17). *x*(*k*) is the *k*th sample of the original signal, and *N*_*s*_ is the sampling number of two adjacent stimulus intervals. $\sqrt{2}$ is the energy matching coefficient, and *y*(*k*) is the filter EMG. In order to make the active intention EMG suitable for trigger control, it needs to be normalized. EMG_*f*_ is the actual electrical amplitude while EMG_0_ is the envelope line. EMG_Max_ is the envelope line in the condition of muscle isometric contraction. *α*presents the active intent coordination coefficient, and its arrange is [0-1]. 0 means no intention output while 1 stands for maximum active power.

(17) $$\begin{array}{c} \alpha =\frac{{EMG}_{f}-{EMG}_{0}}{{EMG}_{\mathrm{Max}}-{EMG}_{0}}. \end{array}$$

## 4. Iterative Learning Control

**Figure 11:**
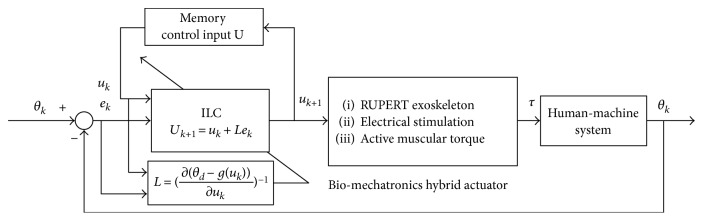
Block diagram of iterative learning control for upper limb rehabilitation system.

Rehabilitation training is a kind of repetitive training. Body state of patients will improve with an increase in the number of training while the auxiliary level of robot and electrical stimulation will be reduced. In addition, it becomes more difficult to control the exoskeleton system because of its highly nonlinear and time-varying characteristics, which are caused in the existence of nonlinear actuators including PMAs and FES. Iterative learning control improves the dynamic system control performance by use of the previous errors and control inputs, which is consistent with the process of rehabilitation training. Patients try to complete the appointed tasks with the help of RUPERT and FES, and the desired trajectory and the actual trajectory will produce movement error. After the one training finishes, the robot returns to the initial position and the motion error information can be used as a prior knowledge of the next training. Iterative learning control is in line with this repetitive training mode shown in Figure 11.

With an increase of assisted rehabilitation trainings, patients' upper limb motor function will improve gradually, so the contribution of patients' active muscular force will increase and the assistance of RUPERT and FES will reduce. Newton's iterative learning control (ILC Newton) is applied in this hybrid rehabilitation system [30, 31]. *k* is the number of iterations, and *θ*_*d*_, *θ*_*k*_, *e*_*k*_, and *u*_*k*_ are the expected angle, the actual angle, the angle error, and the control input of the *k* times iterations, respectively, shown in the following:

(18) $$\begin{array}{c} x_{k}\left(p+1\right)=f\left(x_{k}\left(p\right),u_{k}\left(p\right)\right)={Ax}_{k}\left(p\right)+{Bu}_{k}\left(p\right)\\ \theta_{k}\left(p\right)=h\left(x_{k}\left(p\right)\right)={Cx}_{k}\left(p\right),\;x_{k}\left(0\right)=x_{0}\\ \theta_{k}={\left[\theta_{k}^{T}\left(0\right)\;\theta_{k}^{T}\left(1\right)\;\cdots \;\theta_{k}^{T}\left(T\right)\right]}^{T}\\ u_{k}={\left[u_{k}^{T}\left(0\right)\;u_{k}^{T}\left(1\right)\;\cdots \;u_{k}^{T}\left(T\right)\right]}^{T}\\ \theta_{d}={\left[\theta_{d}^{T}\left(0\right)\;\theta_{d}^{T}\left(1\right)\;\cdots \;\theta_{d}^{T}\left(T\right)\right]}^{T}\\ u_{k+1}=u_{k}+{Le}_{k}\\ e_{k}=\theta_{d}-\theta_{k}\\ \underset{k\to \infty }{\lim}\left\|e_{k}\right\|=0,\underset{k\to \infty }{\lim}\left\|u_{k}-u_{d}\right\|=0\\ \theta \left(0\right)=Cx\left(0\right)=g_{0}\left(x\left(0\right)\right)\\ \theta \left(1\right)=Cx\left(1\right)=C\left(Ax\left(0\right)+Bf\left(u\left(0\right)\right)\right)\\ =g_{1}\left(x\left(0\right),u\left(0\right)\right)\\ \theta \left(2\right)=Cx\left(2\right)=C\left(Ax\left(1\right)+Bf\left(u\left(1\right)\right)\right)\\ =CA\left(Ax\left(0\right)+Bf\left(u\left(0\right)\right)\right)+CBf\left(u\left(1\right)\right)\\ =g_{2}\left(x\left(0\right),u\left(0\right),u\left(1\right)\right)\\ \vdots \\ \theta \left(N-1\right)=Cx\left(N-1\right)=C\left(Ax\left(N-2\right)+Bf\left(u\left(N-2\right)\right)\right)\\ =g_{N-1}\left(x\left(0\right),u\left(0\right),u\left(1\right),\ldots ,u\left(N-2\right)\right)\\ \theta =g\left(\cdot \right)={\left[g_{0}\left(\cdot \right),g_{1}\left(\cdot \right),g_{2}\left(\cdot \right),\ldots ,g_{N-1}\left(\cdot \right)\right]}^{T}. \end{array}$$

Newton method is well known for searching approximate real roots of nonlinear functions through successive approximation, and the specific process is to develop the real valued function *θ*_*d*_ − *g*(*u*_*k*_) by using the Taylor series. By selecting a few terms of the Taylor approximation series of real valued functions and using iterative method for solving *θ*_*d*_ − *g*(*u*_*k*_) = 0, approximate roots are gotten. Given a function *θ*_*d*_ − *g*(*u*_*k*_) and its derivative *g*′(*u*_*k*_), the root of the iterative estimation is shown in the following equations. *u*_*k*_ is the iteration estimates of *k* times, and *u*_*k*+1_ is the iteration estimates of the *k* + 1 times. Newton method converges fast, because the mathematical expression of its convergence rate is the second order rather than linear, and the premise is the existence of inverse *g*′(*u*_*k*_) that means the existence of *g*′(*u*_*k*_)^−1^.

(19) $$\begin{array}{c} u_{k+1}=u_{k}-\frac{\theta_{d}-g\left(u_{k}\right)}{g^{\prime }\left(u_{k}\right)}\\ L={\left(\frac{\partial \left(\theta_{d}-g\left(u_{k}\right)\right)}{\partial u_{k}}\right)}^{-1}=-g^{\prime }{\left(u_{k}\right)}^{-1}\\ u_{k+1}=u_{k}-g^{\prime }{\left(u_{k}\right)}^{-1}e_{k}\\ \Delta u_{k+1}=u_{k+1}-u_{k}\\ e_{k}=-g^{\prime }\left(u_{k}\right)\;\cdot \;u_{k+1}. \end{array}$$

## 5. Experiments of Reach-to-Grasp

**Figure 12:**
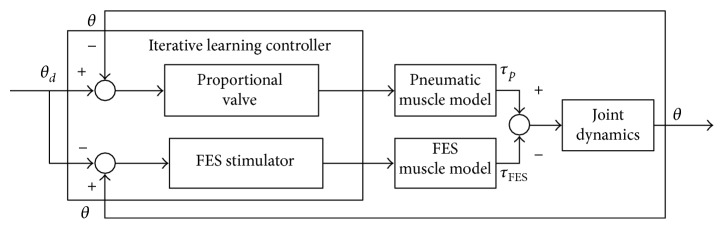
Control block diagram of RUPERT and FES hybrid actuators.

When the angle error *θ*_*d*_ − *θ* of elbow joint is more than zero, the error value is set to *θ*_FES_. When the angle error *θ*_*d*_ − *θ* of elbow joint is less than zero, the error value is set to *θ*_*p*_ which shown in (20). The elbow joint motion is controlled by the proportional valve and FES by use of iterative learning controller as shown in Figure 12.

(20) $$\begin{array}{c} \left[\begin{array}{l} \;\theta_{p}\\ \theta_{FES} \end{array}\right]=\left[\begin{array}{l} -1\\ +1 \end{array}\right]\left[\theta_{d}-\theta \right]. \end{array}$$

*B*_elbow_, *K*_elbow_, and *M*_elbow_ are damping coefficient, stiffness coefficient, and inertia coefficient of elbow joint, respectively. *G*_elbow_ is the gravity moment of elbow joint, and Δ*τ* is the active muscular torque and other bounded disturbances. *τ*_*p*_ and *τ*_FES_ are the torques the pneumatic muscle and FES produce in (21), (22), and (23), respectively. *F*_*p*_, *K*_*p*_, and *B*_*p*_ are the contraction coefficient, stiffness coefficient, and damping coefficient of pneumatic muscle, respectively. *x*_*p*_ is the pneumatic muscle contraction length, and *r*  is the radius of elbow joint. *B*_*p*_ and *B*_FES_ are the torque coefficients of the pneumatic muscle and FES, respectively. $F_{l,v}\left(\theta ,\dot{\theta }\right)$ is the effect of the elbow angle and angular velocity on the FES-induced torque shown in (24). *M*_elbow_, *B*_elbow_, and *K*_elbow_ are 0.02, 0.03, and 0.01, respectively. In order to facilitate the calculation, Δ*τ* is set to zero and the linearization of *G*_elbow_ is equal to 0.02*θ*. Equation (25) is the expression combination of elbow joint drivers. The sampling time *T*_*s*_ is 1 millisecond. *u*_*p*_ and *u*_FES_are the control inputs of pneumatic muscle and FES, respectively, both of which use P-type iterative learning control to update the input. In the process of iterative learning control, when the angle error is within 2 degrees, the iterative process is stopped.

(21) $$\begin{array}{c} \left[\begin{array}{l} \dot{\theta }\\ \ddot{\theta } \end{array}\right]=\left[\begin{array}{l} 01\\ \frac{-K_{elbow}}{M_{elbow}}\frac{-B_{elbow}}{M_{elbow}} \end{array}\right]\left[\begin{array}{l} \theta \\ \dot{\theta } \end{array}\right]+\left[\begin{array}{l} 0\\ \frac{-G_{elbow}+\Delta \tau }{M_{elbow}} \end{array}\right]+\left[\begin{array}{l} 0\\ \frac{B_{i}}{M_{elbow}} \end{array}\right]u_{i},\;i=p,FES \end{array}$$

(22) $$\begin{array}{c} \tau_{p}=B_{p}u_{p},\tau_{FES}=B_{FES}u_{FES} \end{array}$$

(23) $$\begin{array}{c} \tau_{p}=\left(F_{p}\left(u_{p}\right)-K_{p}\left(u_{p}\right)x_{p}-B_{p}\left(u_{p}\right){\dot{x}}_{p}\right)r \end{array}$$

(24) $$\begin{array}{c} \begin{array}{l} \tau_{FES}=\left(\beta_{1}\left|u_{FES}-150\right|{}^{3}+\beta_{2}+\beta_{3}u_{FES}+\beta_{4}u_{FES}^{2}+\beta_{5}u_{FES}^{3}\right)\times \frac{q^{-1}\left(b_{0}+b_{1}q^{-1}\right)}{1+a_{1}q^{-1}+a_{2}q^{-2}}\times F_{l,v}\left(\theta ,\dot{\theta }\right) \end{array} \end{array}$$

(25) $$\begin{array}{c} \left[\begin{array}{l} \dot{\theta }\\ \ddot{\theta } \end{array}\right]=\left[\begin{array}{l} 01\\ -0.48-1.5 \end{array}\right]\left[\begin{array}{l} \theta \\ \dot{\theta } \end{array}\right]+\left[\begin{array}{l} 0\\ \frac{B_{i}u_{i}}{0.02} \end{array}\right]\;i=p,FES. \end{array}$$

This experiment uses the Simulink xPC target real-time control platform, communicating with the surface electrical stimulator Rehastim2 through RS232 serial port to achieve the real-time control. The sampling frequency of xPC target real-time system is 20 kHz, and a PCI-6229 NI acquisition card can output 4-channel DA and control 4-way electromagnetic proportional valves. Pressure signal, force sensor signal, and absolute angle sensors are feedback to the real-time system through the PCI-6229 AD acquisition card. Incremental angle sensors are feedback to the control system through NI PCI-6602. Shoulder joint and elbow joint target angles were *θ*_*d*_(*t*) = [60° 45°]. The task time was *T* = 10 seconds, and RMS was less than 2 degrees.

**Figure 13:**
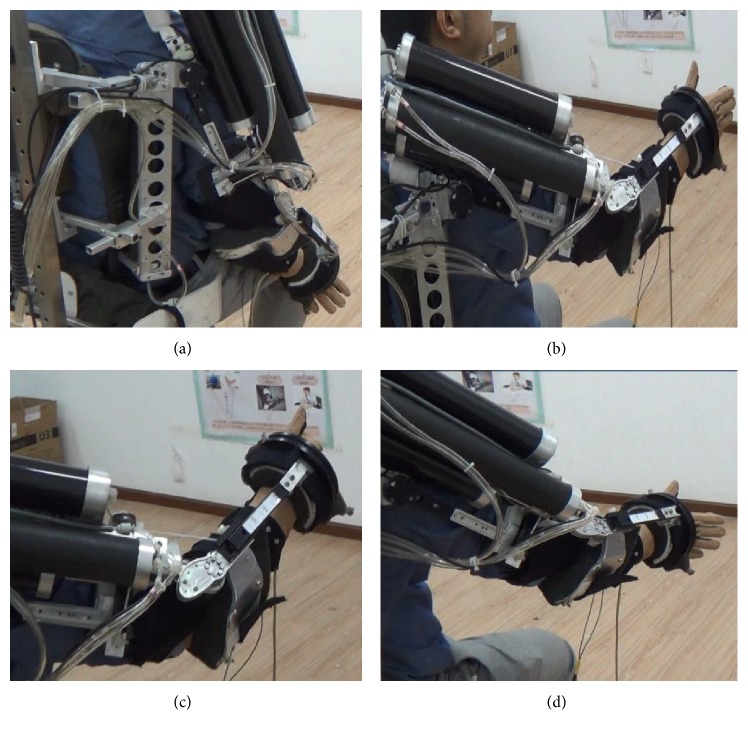
(a) Stretch and grip preparation stages, (b) stretching (ascending process), accompanied by hand release, (c) to achieve the intended goal, hand grip, and (d) stretch (descent process).

After the approval of the ethics committee of Huazhong University of Science and Technology, three healthy subjects were recruited to the treadmill-based exoskeleton gait training experiments (subject 1, male, 32 years old; subject 2, female, 34 years old; and subject 3, male, 29 years old). Before the trainings began, three subjects were informed of the experimental content and purpose shown in Figure 13. In the process of robot and FES-assisted trainings, the subjects were asked to relax as much as possible. The task time is *T* for grasping, and active coordination intention parameter is *α*. In order to reduce the experimental difficulty, the external/internal rotation was fixed to 30 degrees, and only the shoulder and elbow joints were executed. When *α* is less than 0.3, it means hand releasing. When *α* is more than 0.3, it means hand grasping. Stimulus intensity is calculated according to (26). For the above three subjects, *k*_1_ and *k*_2_ were set as 14 and 43, 13 and 43, 17 and 47, respectively.

(26) $$\begin{array}{c} \begin{array}{l} {PA}_{FPL}=k_{1}\times \left(\alpha -0.3\right),\;\alpha >0.3\\ {PA}_{EDC}=k_{2}\times \alpha ,\;\alpha <0.3. \end{array} \end{array}$$

## 6. Experimental Results

**Figure 14:**
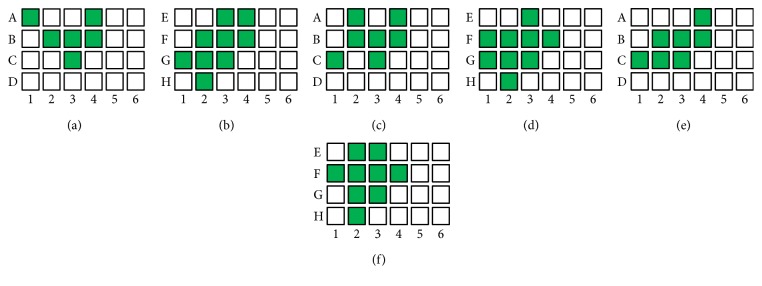
(a) Hand grasping under FES array electrode for subject 1, (b) hand releasing under FES array electrode for subject 1, (c) hand grasping under FES array electrode for subject 2, (d) hand releasing under FES array electrode for subject 2, (e) hand grasping under FES array electrode for subject 3, and (f) hand releasing under FES array electrode for subject 3.

For the same desired hand posture, mapping targets of the three subjects' grasping and releasing under array electrical stimulation are different, shown in Figure 14. Grasping targets of subject 1 are a total of 6 targets with stimulation current amplitude of *I* = 10 m*A*, A1, A4, B2, B3, B4, and C2, respectively. Releasing targets of subject 1 are a total of 9 targets with stimulation current amplitude of *I* = 13 m*A*, E3, E4, F2, F3, F4, G1, G2, G3, and H2, respectively. Grasping targets of subject 2 are a total of 7 targets with stimulation current amplitude of *I* = 9 m*A*, A2, A4, B2, B3, B4, C1, and C3, respectively. Releasing targets of subject 2 are a total of 9 targets with stimulation current amplitude of *I* = 13 m*A*, E3, F1, F2, F3, F4, G1, G2, G3, and H2, respectively. Grasping targets of subject 3 are a total of 7 targets with stimulation current amplitude of *I* = 12 m*A*, A4, B2, B3, B4, C1, C2, and C3, respectively. Releasing targets of subject 3 are a total of 9 targets with stimulation current amplitude of *I* = 14 m*A*, E2, E3, F1, F2, F3, F4, G2, G3, and H2, respectively. About hand grasping and releasing experiments, it can be concluded that each subject's stimulus threshold currents are not the same. These differences are caused by several aspects, including arm morphology, locations of array electrode placement, neuromuscular activation depth, and so on.

**Figure 15:**
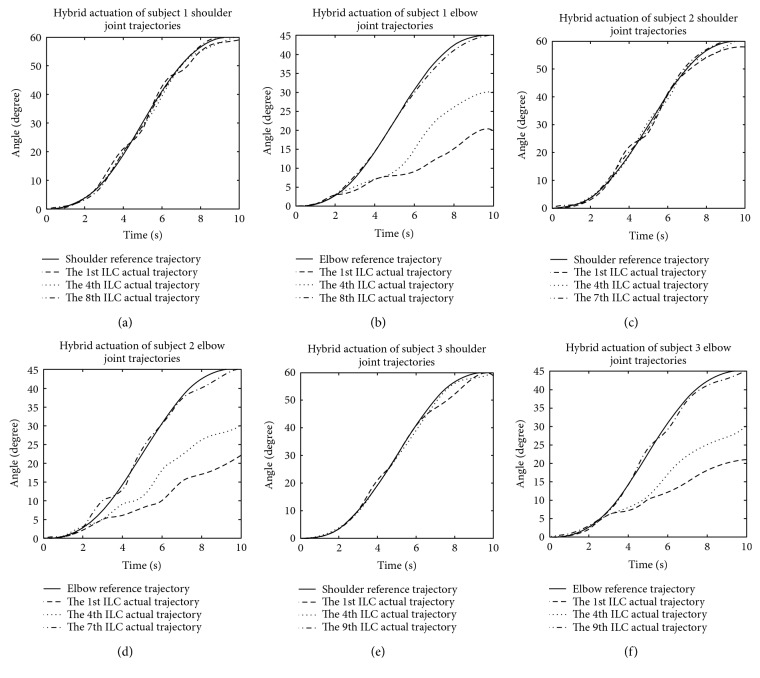
Joint trajectory tracking diagrams of grasping and releasing under FES, (a) shoulder joint for subject 1, (b) elbow joint for subject 1, (c) shoulder joint for subject 2, (d) elbow joint for subject 2, (e) shoulder joint for subject 3, and (f) elbow joint for subject 3.

From Figure 15, it shows that the tracking errors of shoulder and elbow joint movement become smaller and smaller with the increasing of times. For the same error performance indicator, the number of iterations of three subject is 7 times, 8 times, and 9 times, respectively. This difference in the number of iterations may be caused by individual variations of the subjects.

**Figure 16:**
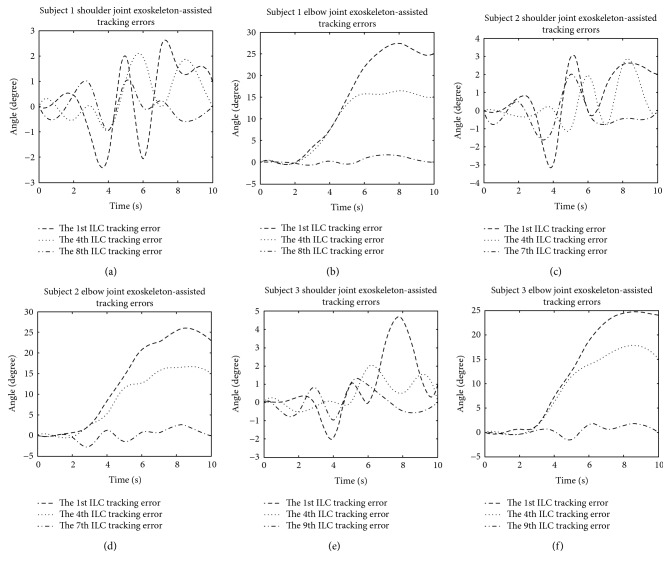
Joint trajectory tracking error diagrams of grasping and releasing under FES, (a) shoulder joint for subject 1, (b) elbow joint for subject 1, (c) shoulder joint for subject 2, (d) elbow joint for subject 2, (e) shoulder joint for subject 3, and (f) elbow joint for subject 3.

Figure 16 shows the angle errors of trajectory tracking in the process of iterations. The tracking errors of shoulder joint have not changed obviously. The elbow joint errors greatly vary between the first time and the last time, and finally the error gradually decreases.

**Figure 17:**
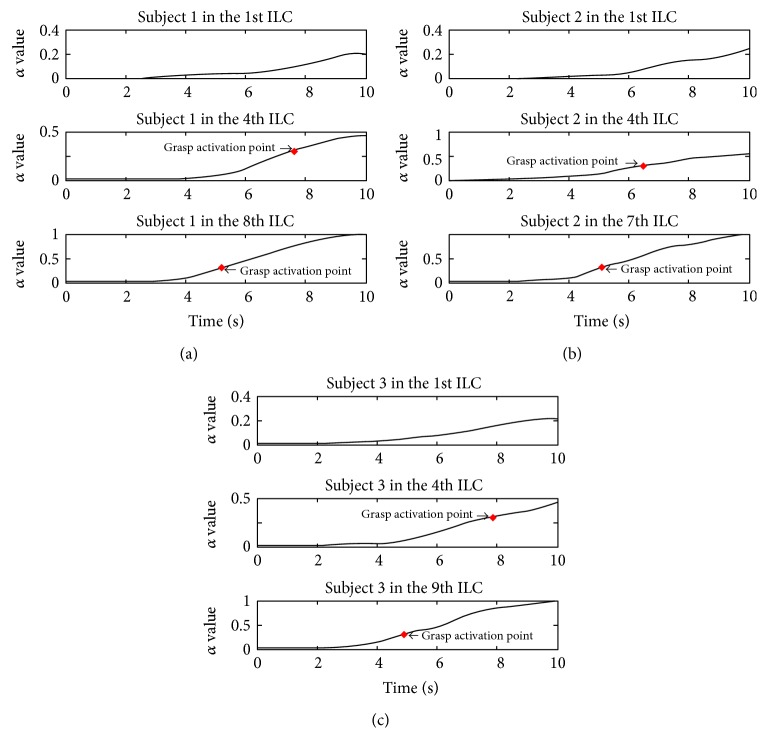
(a) The first subject extended grip coordination training, (b) the second subject extended grip coordination training, and (c) the third subject extended grip coordination training.

As is shown in Figure 17, for all the three subjects in the first iteration of the training process, intention-based grasping task is not activated, which indicates that it may be related to the adaptability of experiments. When it is in the fourth iteration of the training process, grasping intention is detected and grasping task of each subject is activated, but the duration that each subject spent was different. The last iteration is compared with the fourth iteration, which indicates that grasping movement is activated in advance for each subject. This adaptation to intention-based rehabilitation training can help patients to actively participate in trainings and promote the motor function rehabilitation.

## 7. Conclusions and Future Work

In this research, it is presented that upper limb rehabilitation robot powered by PAMs cooperates with FES arrays to realize active reach-to-grasp trainings. FES is taken to activate paralyzed muscles and achieve two-way joint movement targeted for reaching trainings. Modeling of PMA and neuromuscular system under FES and ILC methods is used. The array electrode by virtue of matrix scanning method can solve the problem of the traditional self-adhesive electrode which is time consuming in searching optimum stimulation target. Intention-based FES actively activates the muscles to produce movement. The experimental results validated the effectiveness of this hybrid rehabilitation of robot and FES to realize active reach-to-grasp trainings.

In consideration of their own characteristics of stroke subjects, our proposed integrative strategy is using RUPERT exoskeleton with FES electrically evoked paralyzed ankle muscles to realize reach-to-grasp trainings, which is a promising approach to alleviate the size and mechanical complexity of the robot, thereby the cost of the rehabilitation robot. The future research is discussed for design principle of how to take advantage of each technique in developing a more functional effective hybrid FES and robot-assisted system for upper limb rehabilitation trainings.

## Figures and Tables

**Table 1:** Three-element model of pneumatic muscle model identification parameters.

Coefficient	Contraction		Stiffness		Damping (inflation)		Damping (deflation)	
PAM	*F*_0_	*F*	*K*_0_	*K*_1_	*B*_0_	*B*_1_	*B*_0_	*B*_1_
Shoulder flexion	269.5	1.71	8.65	0.0505	1.31	0.008	0.68	0.0009
Humeral rotation	130.3	0.98	6.11	0.0295	0.88	0.005	0.48	0.0006
Elbow extension	160.7	1.23	6.56	0.0341	0.98	0.006	0.53	0.0007
Forearm pronation	120.3	0.86	5.48	0.0265	0.76	0.004	0.42	0.0005
Wrist extension	115.1	0.81	5.73	0.0243	0.73	0.004	0.39	0.0005

**Table 2:** The identification value of neuromuscular electrical stimulation.

Parameter	Corresponding parameters of muscle model								
	*β*_1_	*β*_2_	*β*_3_	*β*_4_	*β*_5_	*a*_1_	*a*_2_	*b*_0_	*b*_1_
Value	2.41 × 10^−8^	−0.0294	0.0023	−6.98 × 10^−6^	1.56 × 10^−8^	−1.21	0.117	0.1	0
